# Behavioral Comorbidities and Drug Treatments in a Zebrafish *scn1lab* Model of Dravet Syndrome

**DOI:** 10.1523/ENEURO.0066-17.2017

**Published:** 2017-08-14

**Authors:** Brian P. Grone, Tiange Qu, Scott C. Baraban

**Affiliations:** 1 Epilepsy Research Laboratory in the Department of Neurological Surgery, University of California, San Francisco, CA; 2 Helen Wills Neuroscience Institute, University of California, Berkeley, CA; 3 Weill Institute for Neurosciences, University of California, San Francisco, San Francisco, CA 94143

**Keywords:** anxiety, Dravet syndrome, epilepsy, sleep, sodium channels, zebrafish

## Abstract

Loss-of-function mutations in *SCN1A* cause Dravet syndrome (DS), a catastrophic childhood epilepsy in which patients experience comorbid behavioral conditions, including movement disorders, sleep abnormalities, anxiety, and intellectual disability. To study the functional consequences of voltage-gated sodium channel mutations, we use zebrafish with a loss-of-function mutation in *scn1lab*, a zebrafish homolog of human *SCN1A*. Homozygous *scn1lab*^s552/s552^ mutants exhibit early-life seizures, metabolic deficits, and early death. Here, we developed *in vivo* assays using *scn1lab*^s552^ mutants between 3 and 6 d postfertilization (dpf). To evaluate sleep disturbances, we monitored larvae for 24 h with locomotion tracking software. Locomotor activity during dark (night phase) was significantly higher in mutants than in controls. Among anticonvulsant drugs, clemizole and diazepam, but not trazodone or valproic acid, decreased distance moved at night for *scn1lab* ^s552^ mutant larvae. To monitor exploratory behavior in an open field, we tracked larvae in a novel arena. Mutant larvae exhibited impaired exploratory behavior, with increased time spent near the edge of the arena and decreased mobility, suggesting greater anxiety. Both clemizole and diazepam, but not trazodone or valproic acid, decreased distance moved and increased time spent in the center of the arena. Counting inhibitory neurons *in vivo* revealed no differences between *scn1lab* ^s552^ mutants and siblings. Taken together, our results demonstrate conserved features of sleep, anxiety, and movement disorders in *scn1lab* mutant zebrafish, and provide evidence that a zebrafish model allows effective tests of treatments for behavioral comorbidities associated with DS.

## Significance Statement

Certain mutations cause severe genetic diseases that affect brain development, leading to seizures, cognitive impairment, and abnormal behaviors in affected children. One of the best studied of these genetic diseases is Dravet syndrome (DS), which results from mutations in a channel that normally controls sodium flux in the brain. Although the genetic cause of DS is known, no effective treatment is available. Animals, including zebrafish, share sodium channels that are similar to the human ones, and mutating a sodium channel also leads to an epilepsy disorder in developing zebrafish. We found that zebrafish with a DS-like mutation also exhibit problems with locomotion, sleep, and anxiety, and that these behaviors were modulated by antiepileptic drugs.

## Introduction

Voltage-gated sodium channels are responsible for generation and propagation of neuronal action potentials in central and peripheral nervous systems. Mutations of these channels are a major cause of genetically inherited epilepsies and other neurologic disorders (Dichgans et al., 2005; Meisler and Kearney, 2005; Catterall, 2014; Waxman et al., 2014). *SCN1A*, which encodes the pore-forming alpha subunit of Na_v_1.1, is expressed throughout mammalian nervous systems, primarily in axon initial segments and nodes of Ranvier (Gordon et al., 1987; Duflocq et al., 2008). Confirming the conserved importance of Na_v_1.1 for neural function, homologs of *SCN1A* are present in other vertebrates, including teleost fishes (Novak et al., 2006a,b; Widmark et al., 2011).

Nonsense and missense mutations in *SCN1A* are associated with a catastrophic epilepsy of childhood known as Dravet syndrome (DS; Escayg et al., 2000; Claes et al., 2001; Sugawara et al., 2002; Harkin et al., 2007; Depienne et al., 2009; Dravet, 2011; Catterall, 2014). In DS, seizures commonly appear in the first year of life (often accompanied by fever), and progress to prolonged myoclonic, atypical absence, or focal events with frequent status epilepticus episodes requiring emergency care (Gataullina and Dulac, 2017). Generalized and multifocal abnormalities are seen in the electroencephalography. Starting as early as the second year of life, DS children develop comorbid conditions such as psychomotor regression, motor disorder, abnormal sleep microarchitecture, and intellectual disability (Nolan et al., 2006; Martin et al., 2010; Dhamija et al., 2014). The risk for premature death is also high in this patient population (Genton et al., 2011). Unfortunately, effective treatments are not available to address the range of seizure phenotypes and comorbidities associated with DS (Chiron and Dulac, 2011; Catterall, 2014; Wilmshurst et al., 2014). Studies in animal models, which now include zebrafish as well as mice, offer a means to address this problem (Catterall, 2014; Grone and Baraban, 2015; Kaplan et al., 2016).

Mice with heterozygous deletion of *Scn1a* reproduce many DS phenotypes, including epilepsy with early onset (Yu et al., 2006; Ogiwara et al., 2007), susceptibility to febrile seizures (Oakley et al., 2009), sleep and circadian abnormalities (Han et al., 2012a; Papale et al., 2013), and premature death (Kalume et al., 2013). Reduced neuronal excitability and behavioral abnormalities are also found in *Scn1a* mutant mice (Han et al., 2012b; Ito et al., 2013). Although mice offer strengths for understanding the basic biology and physiology of ion channels and for testing treatments, they are not ideally suited to drug discovery.

Zebrafish have emerged as a powerful model organism for analyzing genetic diseases (Ablain and Zon, 2013; Deciphering Developmental Disorders Study, 2015; Grone et al., 2016; Tuschl et al., 2016). Zebrafish with a missense loss-of-function mutation in *scn1lab*, one of two *SCN1A-*like genes found in teleost fishes (Novak et al., 2006b), show oculomotor defects, early life seizures, pharmacoresistance, and metabolic deficits (Schoonheim et al., 2010; Baraban et al., 2013; Kumar et al., 2016; Sourbron et al., 2016). Like *Scn1a* null mice, which develop ataxia and die at about postnatal day 15 (Yu et al., 2006; Ogiwara et al., 2007), homozygous *scn1lab* ^s552/s552^ mutant larvae do not survive beyond 14 d postfertilization (dpf; Schoonheim et al., 2010). This well-conserved overall phenotype suggests that the *scn1lab* gene in zebrafish shares essential conserved functions with mammalian *Scn1a*. Whether *scn1lab* mutant zebrafish exhibit comorbidities normally associated with DS, including movement disorders, anxiety, or sleep disturbances, is not known. Here, we provide the first assessments of these behaviors in a zebrafish model of DS, i.e., *scn1lab* ^s552^ mutants. We used a set of assays based on zebrafish sleep patterns (Zhdanova et al., 2001; Yokogawa et al., 2007; Rihel et al., 2010), and behavioral responses to novel environments (Richendrfer et al., 2012; Schnörr et al., 2012; Ahmad and Richardson, 2013). We found that the homozygous *scn1lab* ^s552^ mutants exhibit nighttime hyperactivity, decreased time spent in the center of an open arena, and decreased responsiveness to sudden darkness. Diazepam and clemizole have previously been found to suppress convulsive seizure behaviors in this model (Baraban et al., 2013; Griffin et al., 2017). We found that both of these drugs also reduced nighttime locomotor activity and the time spent in the center of the open field. Taken together, our results suggest that behavioral comorbidities can be studied in larval zebrafish models of genetic epilepsies, and that these mutant fish could aid in identifying new treatments.

## Materials and Methods

### Zebrafish maintenance

Adult male and female zebrafish were maintained according to standard procedures (Westerfield, 2000) and following guidelines approved for this study by the University of California, San Francisco Institutional Animal Care and Use Committee. The *Tg(1.4dlx5a-dlx6a:GFP)* fish line has been previously described (Ghanem et al., 2003) and was generously provided by the laboratory of Dr. Marc Ekker. The *scn1lab*^s552^ line has been previously described (Schoonheim et al., 2010) and was generously provided by the laboratory of Dr. Herwig Baier. Zebrafish of the TL strain were obtained from the Zebrafish International Resource Center (ZIRC). The zebrafish room was maintained on a 14/10 h light/dark cycle, with lights-on at 9 A.M. and lights-off at 11 P.M. Fish system water conditions were maintained in the following ranges by automated feedback controls: 29–30°C, pH 7.5–8.0, conductivity (EC) 690–710. Zebrafish embryos and larvae were raised in an incubator maintained at 28.5°C, on the same light-dark cycle as the fish facility. At 5 dpf, zebrafish have not yet experienced sexual differentiation (Liew and Orbán, 2014). Water used for embryos and larvae (“embryo medium”) was made by adding 0.03% Instant Ocean and 0.000002% methylene blue to reverse-osmosis distilled water. Embryos and larvae were raised in plastic Petri dishes (90 mm in diameter, 20 mm in depth) and their housing density was limited to ∼60 individuals per dish.

### Pharmacology

The following drugs were dissolved in dimethylsulfoxide (DMSO, Sigma-Aldrich) to 10 mM as stock solutions, and stored at −20°C until needed: caffeine (Sigma), clemizole-HCl (Tocris), diazepam (Sigma-Aldrich), trazodone-HCl (Sigma-Aldrich), and valproic acid sodium salt (Sigma-Aldrich).

### Behavioral phenotyping, diurnal activity

**Figure 1.**
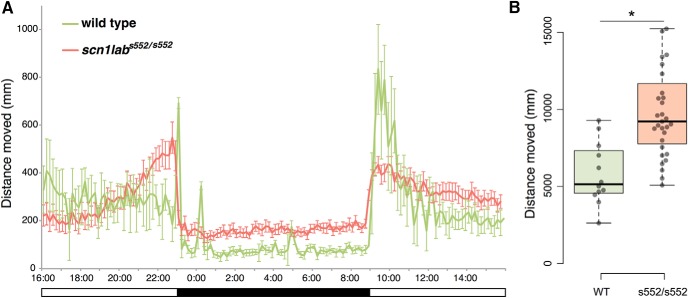
Mutant *scn1lab*^s552^ larvae had statistically significantly more locomotor activity at night compared to wild-type (WT) siblings. Larval zebrafish (5 dpf) were placed in individual wells of a flat-bottom 96-well plate and acclimated to the recording chamber. Twenty-four hours of movement data were collected beginning at 4 P.M. ***A***, Data shown are sums of 10-min bins ± SD (*N* = 12 WT, 31 Mut). The dark bar at the bottom indicates the 10-h period when lights were turned off, 11 P.M. to 9 A.M. ***B***, Total nighttime distance moved by WT and homozygous mutant (s552/s552) larvae. Boxplot shows median, quartiles, and whiskers extending to the furthest point within 1.5 IQR (dot plots are shown, with outliers excluded). *p < 0.05.

To monitor diurnal activity patterns, *scn1lab* ^s552/s552^ larvae were placed individually in wells of a flat-bottom 96-well Falcon culture dish (BD Biosciences) and movement was tracked continuously during a 24-h period, which included 14 h light-on and 10 h light-off phases. Each well contained ∼200 μl of embryo medium. Behavior was monitored at room temperature (21–22°C) using two DanioVision systems and EthoVision XT locomotion tracking software version (Noldus). The older system with EthoVision XT 8 software was used for initial experiments (Fig. 1). The newer system with EthoVision XT 11 software was used for all other experiments. Total distance moved measurements were consistent within systems, but not comparable between systems as the software detection parameters and image quality are not identical on the two systems; 5-dpf larvae were allowed to acclimate to the tracking arena for 3–4 h, and then 24 h of continuous behavioral data were recorded beginning at 4 P.M. The light-dark cycle continued as usual: lights-off occurred at 11 P.M. and lights-on at 9 A.M.

### Behavioral phenotyping, open field

Open field behavior was examined in wells of a six-well plate, each containing 6 ml of egg water; 5-dpf zebrafish larvae were placed individually in separate wells, using a glass Pasteur pipette. Larvae are initially placed near the center of the arena, but tend to shift slightly as the plate is transferred into the recording apparatus. Using EthoVision, 5 min of tracking data were immediately recorded with no acclimation period. The video data were analyzed in 30-s time bins. For each group, we calculated distance traveled and time spent in the center zone (diameter = 25.5 mm) of the well (inner diameter = 36.6 mm).

For drug treatment trials, drugs in DMSO were diluted in embryo medium to a final concentration of 250 μM, 25 μM, or 2.5 μM, as described (with 2.5% DMSO). Zebrafish larvae were incubated in embryo medium containing the drug or DMSO for 30 min before transfer to the open field (in groups of three fish in 2 ml of solution in a well of a 24-well plate). Individual larvae were then transferred to a single well of a six-well plate containing DMSO (2.5%) or drug dissolved in DMSO, for the duration of the assay.

### Cell count

For imaging of interneurons, we in-crossed *scn1lab*^s552/+^;*Tg(1.4dlx5a-dlx6a:GFP)* adult zebrafish. Green fluorescent protein (GFP)-expressing larvae were raised in PTU and imaged at 5 dpf using a Zeiss Z.1 light sheet microscope with 20× objective. The sample chamber was filled with embryo medium. Zebrafish larvae were anesthetized in 0.04% tricaine mesylate for 10 min, then embedded in 2% low melting point agarose inside a glass capillary. Image stacks were taken extending from the first dorsal GFP-positive neuron, at intervals of 4.94 μm with 20 slices per fish. Imaging files were coded and analyzed *post hoc* by an investigator blind to phenotype and genotype. Following imaging, the fish were removed from agar and genotyped.

FIJI software was used for analysis of image stacks (Schindelin et al., 2012). Cells were counted automatically using 3D Objects Counter.

### Genotyping

For genotyping, we extracted genomic DNA (gDNA) from whole larvae using the Zebrafish Quick Genotyping DNA Preparation kit (Bioland Scientific). We amplified *scn1lab* gDNA using GoTaq Green Master Mix (Promega) and the following primers: *scn1lab*-F, AAATCTCTCTGTTAGACAGAAATTGGGG and *scn1lab*-R, TTGCTCAGGCTGTGTGATGAGG. These primers amplify a 314-bp region, including the *scn1lab* mutation site. The mutant allele was then detected by digestion of the amplicon with AcuI, for which a restriction site is introduced in the mutant, and electrophoresis to separate the digested samples on a 1% agarose gel.

### Statistics and graphing

JASP version 0.8.0.1 software was used for statistical tests (https://jasp-stats.org/). Microsoft Excel, R software (R Core Team, 2016) and the beeswarm package were used for plotting data.

## Results

### Diurnal locomotor activity is altered in mutant larvae

To assess diurnal movements indicative of sleep/wake cycles, we tracked larvae from *scn1lab* ^s552/+^ in-crosses continuously during a 24-h light/dark period (Zhdanova et al., 2001; Yokogawa et al., 2007; Rihel et al., 2010; Elbaz et al., 2013). To quantify disruptions to sleep activity, total distance moved during the dark (sleep) period was compared between groups. Distance moved at night (11 P.M. to 9 A.M.) showed statistically significant differences between genotypes: *scn1lab* ^s552/s552^ traveled a greater distance compared to wild-type siblings (Fig. 1*B*). Welch’s *t* test showed a difference between genotypes (mean ± SEM, measured in mm): wild type, 5622.7 ± 1181.5 (*N* = 12); homozygous 10,084.0 ± 1244.8 (*N* = 31); *t*_(39.87)_ = 4.410, *p* < 0.001. These differences in diurnal activity patterns suggest that *scn1lab* ^s552/s552^ larvae exhibit sleep and diurnal rhythm disturbances.

In the final hour before lights-off (10–11 P.M.), *scn1lab* ^s552/s552^ mutant larvae traveled a greater distance (2900 ± 327.5) compared to wild-type siblings (1490 ± 339.3; *t*_(31.14)_ = 2.989; *p* = 0.005). We observed trends toward increased activity by the mutants in the first hour of tracking (4-5 P.M.; mutant, 1264 ± 227.8; wild type, 2090 ± 600.3; *t*_(14.29)_ = 1.289, *p* = 0.218), and the first hour after lights-on (9–10 A.M.; mutant, 2341 ± 162.0; wild type, 3715 ± 800.5; *t*_(11.91)_ = 1.683, *p* = 0.118), but these did not reach statistical significance.

### Sleep pharmacology

**Figure 2.**
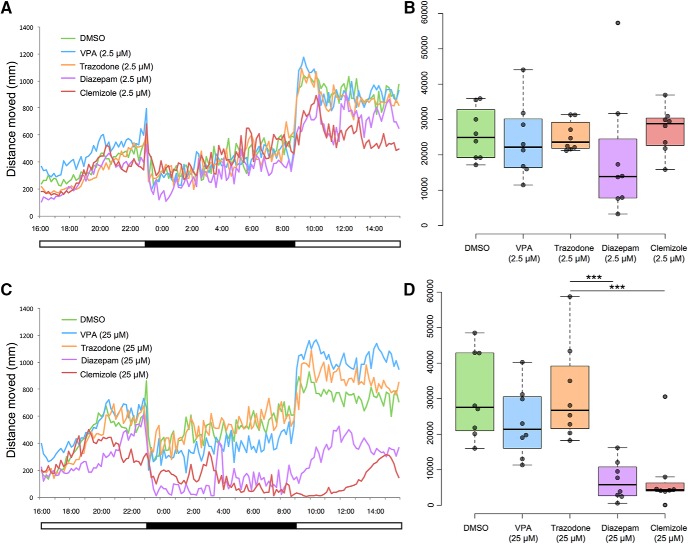
Full 24-h light-dark cycle behavioral data reveal effects of treatment with 2.5 μM (***A***, ***B***) and 25 μM (***C***, ***D***) concentrations of drugs in DMSO. Both diazepam and clemizole, at 25 μM concentration, significantly decreased distance moved at night compared to vehicle (DMSO)-treated control. Mean values (*N* = 8 individuals per group) per 10-min time bin are shown at left (***A***, ***C***). Total night-time distances moved (during 10 h of darkness), are shown at right (***B***, ***D***). Boxplot shows median, quartiles, and whiskers extending to the furthest point within 1.5 IQR (dot plots are shown, with outliers excluded). The 2.5 μM concentration of each drug had no significant effect on total nighttime locomotor activity, an indicator of wakefulness (***B***). On the other hand, the 25 μM treatment with either diazepam or clemizole significantly reduced the nighttime locomotor activity (***D***). ***p < 0.001.

To determine if the observed disruptions of diurnal rhythms could be pharmacologically alleviated, drug trials were conducted on *scn1lab* ^s552/s552^ larvae during a full 24-h period. We found that drug treatments could significantly decrease nighttime locomotor patterns indicative of wakefulness. We tested two drug concentrations based on previous reports (Herrmann, 1993; Zhdanova et al., 2001; Richendrfer et al., 2012; Baraban et al., 2013; Koseki et al., 2014) and pilot experiments; a “low” concentration of 2.5 μM and a “high” concentration of 25 μM for four compounds: valproic acid, diazepam, trazodone, and clemizole (Fig. 2).

At 2.5 μM concentration (Fig. 2*B*), *t* tests showed no difference between DMSO and valproic acid, trazodone, diazepam, or clemizole (Table 1). At 25 μM concentration (Fig. 2*D*), *t* tests showed no difference between DMSO and valproic acid or trazodone. On the other hand, *t* tests revealed that two drug treatments led to less distance traveled compared to DMSO: diazepam and clemizole (Table 1).

**Table 1.** Night phase movement statistical data

Drug	Mean ± SEM	Student’s *t*	df	*p*	Levene’s *p*	Shapiro-Wilk *p*
(2.5 µM)						
DMSO control	25,868.7 ± 2606.4					0.308
Valproic acid	24,120.2 ± 3697.7	0.386	14	0.705	0.432	0.651
Trazodone	25,239 ± 1494.6	0.209	14	0.837	0.109	0.068
Diazepam	19,108.0 ± 6247.0	0.999	14	0.335	0.133	0.034
Clemizole	27,076.0 ± 2289.8	0.348	14	0.733	0.594	0.863
(25 µM)						
DMSO control	(30,921.1 ± 4311.6)					0.293
Valproic acid	23,435.8 ± 3465.6	1.353	14	0.197	0.324	0.75
Trazodone	31,490.0 ± 4857.9	0.088	14	0.931	0.932	0.188
Diazepam	6908.1 ± 1916.6	5.089	14	<0.001	0.01	0.567
Clemizole	7475.7 ± 3384.2	4.278	14	<0.001	0.171	<0.001

Larval zebrafish (*N* = 18 per group) were video recorded during the 10-h night phase and total distance was measured for larva treated with DMSO or with one of four drugs at two concentrations (see Materials and Methods). For DMSO and each drug tested, the table shows distance moved in mm (mean ± SEM), *t* value, degrees of freedom, and *p* value (see Results). Data from lower concentration tested (2.5 µM) is shown at top; 25 µM is shown below.

### Open field deficits in mutant larvae

To study anxiety-like and locomotor behavior in more detail, we adapted a version of the open field test (Walsh and Cummins, 1976). This assay is designed to give temporal as well as spatial resolution regarding position and movement over time after larvae are introduced to a novel cylindrical chamber. Single larvae were placed in individual wells of a flat-bottom six-well plate and movement was tracked during a 5-min recording epoch. DMSO (2.5%) was tested for effects on larval behavior compared to embryo medium using Welch’s *t* test (*N* = 6 per group). No significant effects were found for total duration in center (mean ± SEM): water, 87.9 ± 44.7; DMSO, 116.6 ± 45.2 (*t*_(10.0)_ = 0.451, *p* = 0.662) or total distance moved (mean ± SEM, measured in mm): water, 869.8 ± 69.8; DMSO, 815.2 ±36.0 (*t*_(7.49)_ = 0.695, *p* = 0.508), or any of the individual time bins.

**Figure 3.**
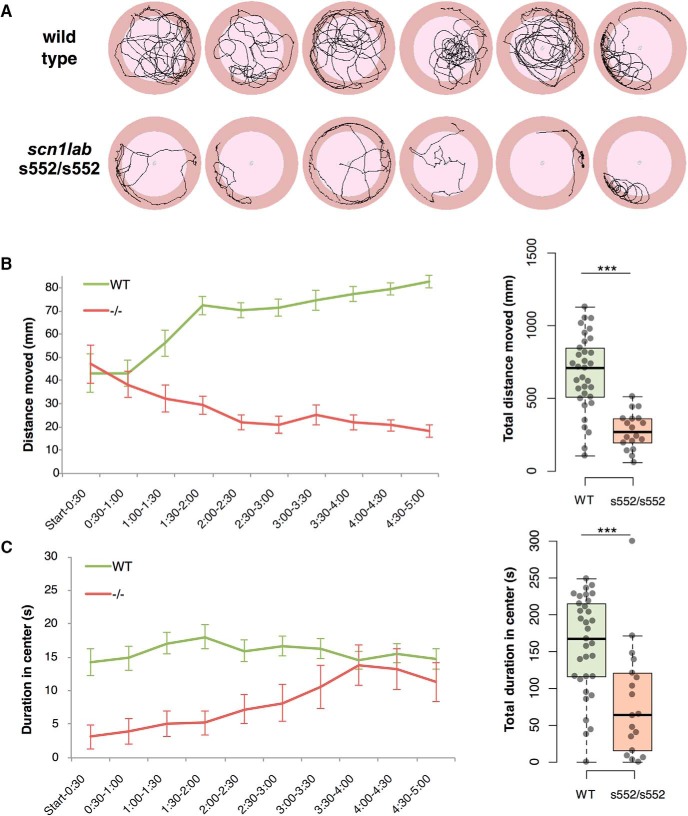
Disruptions in open field test behaviors were found in *scn1lab* ^s552/s552^ larvae. Representative traces of movement are shown (***A***). Mutant *scn1lab* ^s552/s552^ larvae initially have normal levels of overall movement (***B***) but spend less time than controls in the center of the arena (***C***). At subsequent time points, *scn1lab* ^s552/s552^ larvae’s movement decreases (***B***) and their time spent in the center of the arena increases (***C***). Time bins are 30 s, bars show SEM. On the right, dot plots are shown with boxplot indicating median, quartiles, and whiskers extending to the furthest point within 1.5 IQR; statistical analyses were conducted on these totals. ***p < 0.001.

Representative tracking plots for six larvae per genotype are shown in Figure 3. In this assay, *scn1lab* ^s552/s552^ mutant larvae have significantly impaired (low) distance moved compared to wild-type control sibling larvae. Total distance moved (Fig. 3*B*) was different between genotypes (mean ± SEM, measured in mm): wild type, 5622.7 ± 1181.5 (*N* = 33); homozygous, 10,084.0 ± 1244.8 (*N* = 18) by Welch’s *t* test (*t*_(48.43)_ = 7.256, *p* < 0.001). For time spent in the center zone (25.5 mm) of the arena (36.6 mm), homozygous *scn1lab* ^s552/s552^ mutants were significantly reduced compared to wild-type siblings. Duration in center (Fig. 3*C*) was also different between wild-type and homozygous mutants (mean ± SEM): wild type, 158.2 ± 11.6 (*N* = 33); homozygous, 81.7 ± 18.1 (*N* = 18) by Welch’s *t* test (*t*_(31.11)_ = 3.552, *p* = 0.001).

### Open field pharmacology

**Figure 4.**
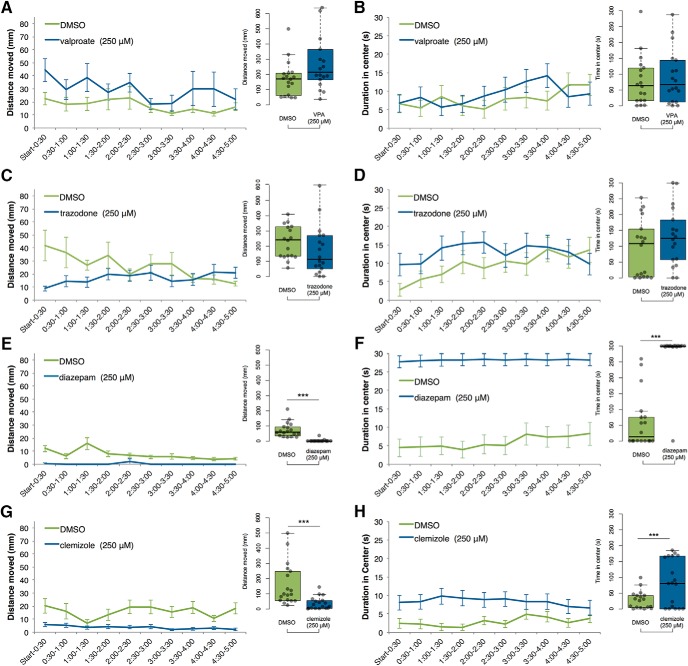
Treatment with antiepileptic drugs at 250 μM. Statistically significant decreases in locomotion (distance traveled) and duration in the center of the open field were observed after treatment of zebrafish larvae with 250 μM diazepam (***C***, ***D***) or clemizole (***G***, ***H***), but not after treatment with valproic acid (***A***, ***B***) or trazodone (***E***, ***F***; *N* = 18 per group). Data are plotted in 30-s time bins showing mean ± SEM (left), and the 5-min total for each all individuals, are plotted on the right. ***p < 0.001.

Next, we pretreated *scn1lab* ^s552/s552^ larvae with drugs and assessed effects using the open field assay. All drugs (valproic acid, diazepam, trazodone, and clemizole) were tested at two different concentrations, 25 μM and 250 μM. The 25 μM concentration of each drug had no significant effect on either time spent in the center or total movement (Fig. 4). No effect was observed after treatment of zebrafish larvae with 25 μM valproic acid, diazepam, trazodone, or clemizole on distance moved or duration spent in the center of the arena in an open field test (*N* = 18 per group). All drug treatments were compared to DMSO vehicle using Welch’s *t* test (Table 2).

**Table 2.** Open field test statistical data

Drug	Measurement	Treated	DMSO control	Welch’s *t*	df	*p*
(25 µM)						
VPA	Distance (mm)	287.6 ± 37.31	234.2 ± 29.23	1.127	32.16	0.268
	Center duration (s)	91.93 ± 16.54	64.51 ± 17.93	1.124	33.78	0.269
Trazodone	Distance (mm)	199.9 ± 29.26	198.0 ± 17.79	0.055	28.05	0.956
	Center duration (s)	72.81 ± 21.17	85.73 ± 19.31	0.451	33.72	0.655
Diazepam	Distance (mm)	138.5 ± 28.87	175.6 ± 26.73	0.941	33.8	0.354
	Center duration (s)	73.25 ± 18.55	66.49 ± 18.03	0.261	33.97	0.796
Clemizole	Distance (mm)	177.9 ± 32.12	107.5 ± 15.12	1.983	24.18	0.059
	Center duration (s)	49.17 ± 18.93	97.65 ± 22.17	1.663	33.19	0.106
(250 µM)						
VPA	Distance (mm)	293.6 ± 52.82	172.2 ± 26.99	2.047	25.31	0.051
	Center duration (s)	91.07 ± 20.05	78.36 ± 18.31	0.468	33.72	0.643
Trazodone	Distance (mm)	168.8 ± 38.14	260.1 ± 43.95	1.57	33.34	0.126
	Center duration (s)	128.82 ± 21.62	93.96 ± 21.30	1.149	33.99	0.259
Diazepam	Distance (mm)	2.56 ± 2.04	73.06 ± 11.40	6.089	18.08	<0.001
	Center duration (s)	282.07 ± 16.59	59.73 ± 20.15	8.518	32.8	<0.001
Clemizole	Distance (mm)	36.89 ± 9.61	158.38 ± 32.62	3.572	19.93	0.002
	Center duration (s)	83.29 ± 17.31	28.31 ± 6.61	2.967	21.85	0.007

Larval zebrafish (*N* = 18 per group) were pretreated one of four drugs at two concentrations (see Materials and Methods). Each treatment group was tested at the same time as a control group treated with DMSO. Drug-treated and control larvae were then placed in a novel arena and behavior was video recorded for 5 min. Both distance moved (mm) and duration spent in center of the arena (s) were measured for all larva. Table shows distance moved in mm (mean ± SEM), *t* value, degrees of freedom, and *p* value. Data from lower concentration tested (25 µM) is shown at top; 250 µM is shown below.

On the other hand, the 250 μM concentration of either diazepam or clemizole, the same two drugs with significant effects in our sleep assay (Fig. 2), significantly reduced the overall locomotor activity we observed while increasing the duration spent in the center of the arena, a measure of low-anxiety exploratory behavior (Fig. 4). All drug treatments were compared to DMSO vehicle using Welch’s *t* test. Valproic acid and trazodone had no significant effect on distance moved or duration in center (Fig. 4*A-D*). Diazepam and clemizole decreased distance moved and increased duration in center (Fig. 4*E-H*).

**Figure 5.**
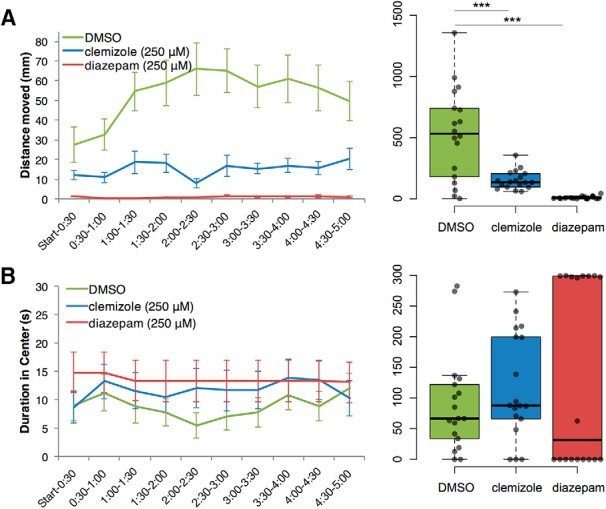
Wild-type TL zebrafish larvae open field behavior is modulated by clemizole and diazepam. Statistically significant decreases in distance traveled (***A***) in open field test were observed after treatment of zebrafish larvae with 250 μM clemizole or diazepam, compared to DMSO-treated controls. No statistically significant effects on duration spent in the center (***B***) were found for either clemizole or diazepam. ***p < 0.001.

The effects of clemizole and diazepam, which significantly modified open field behavior in *scn1lab* ^s552/s552^ larvae, were also tested further in wild-type larvae of the TL strain (Fig. 5). Clemizole and diazepam were compared to DMSO in the same experiment (*N* = 18 per group).

For total distance traveled during the 5-min assay, clemizole (153.3 ± 17.84) and diazepam (10.25 ± 2.76), led to significant reductions compared to the control DMSO-treated larvae (529.0 ± 88.26), using Welch’s *t* test (clemizole: *t*_(18.39)_ = 4.172, *p* < 0.001; diazepam: *t*_(17.03)_ = 5.875, *p* < 0.001). In TL larvae, we found no significant effects of either clemizole (117.0 ± 20.45) or diazepam (136.1 ± 35.4) on total duration in center measured in mm, compared to DMSO (89.1 ± 19.1), using Welch’s *t* test (clemizole: *t*_(33.9)_ = 0.999, *p* = 0.325; diazepam: *t*_(26.2)_ = 1.169, *p* = 0.253).

To further characterize the effects of pharmacological interventions in our open field assay, we tested the effect of caffeine at a concentration of 250 μM (Maximino et al., 2011), using the same protocol as applied for the other drugs. Caffeine-treated wild-type larvae moved significantly less (mean ± SEM, measured in mm; 333.4 ± 56.23) than DMSO-treated controls (562.5 ± 105.0; *t*_(32)_ = 2.103, *p* < 0.043), but did not significantly differ in duration spent in the center of the arena (*t*_(32)_ = 0.679, *p* < 0.465). In the mutant larvae, we observed no significant effects of caffeine on either total distance traveled (*t*_(37)_ = 1.428, *p* < 0.162) or duration spent in the center of the arena (*t*_(37)_ = 0.425, *p* = 0.673).

### Interneuron density

**Figure 6.**
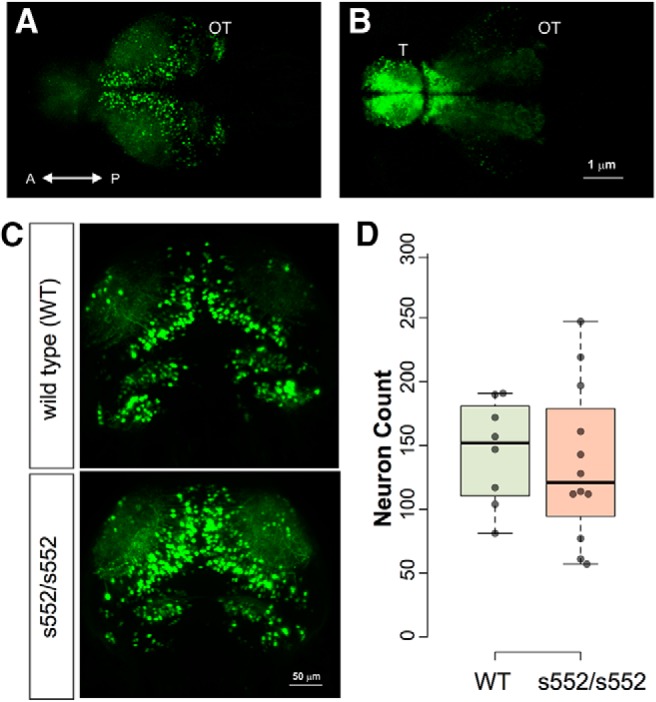
No significant effects of *scn1lab* mutation were found in numbers of *Tg(1.4dlx5a-dlx6a:GFP)* neurons. Neurons were counted by light-sheet microscopy in 5-dpf larvae, followed by 3D image segmentation and quantification of discrete objects. Sample images are shown illustrating GFP fluorescence detected in optic tectum (***A***) and telencephalon (***B***). Representative images (***C***) are shown for wild-type and *scn1lab* ^s552/s552^ mutants. Quantification of cell numbers is shown in (***D***) as dot plots with boxplot indicating median, quartiles, and whiskers extending to the furthest point within 1.5 IQR. No significant effect of genotype was detected.

Because mutations in mammalian *Scn1a*, which is expressed in inhibitory interneurons, impair interneuron firing activity (Yu et al., 2006; Mistry et al., 2014), we examined interneuron numbers at 5 dpf. To visualize interneurons *scn1lab* ^s552/s552^ were crossed with a zebrafish line expressing a transgene with GFP flanked by both a 3.5-kb fragment of the *dlx6* promoter and a 1.4-kb fragment of the *dlx5/6* intergenic region (Ghanem et al., 2003). This reporter line labels distinct populations of interneurons (Robles et al., 2011). To evaluate interneuron density we conducted live light-sheet microscopy of *scn1lab*^s552/s552^;*Tg(1.4dlx5a-dlx6a:GFP)* larvae (Fig. 6). Live imaging revealed cells labeled by GFP in the telencephalon, optic tectum, cerebellum, and diencephalon. In an imaging stack centered on the optic tectum, we counted GFP-positive cells for *scn1lab* ^s552/s552^ and sibling larvae. Welch’s *t* test was used to test for differences between genotypes (mean ± SEM): wild type, 144.9 ± 11.7 (*N* = 8); homozygous, 135.7 ± 17.5 (*N* = 12). No statistically significant effect of genotype was found (*t*_(17.99)_ = 0.406, *p* = 0.690).

## Discussion

Behavioral deficits greatly decrease quality of life for DS patients. Our findings here demonstrate that zebrafish provide useful models of behavioral as well as neurophysiological symptoms of epileptic encephalopathies such as DS. While *Scn1a* haploinsufficiency is known to cause sleep and circadian abnormalities, hyperactivity, autistic- and anxiety-like behavior in mouse models, these comorbidities have not been previously investigated in a systematic way in *scn1lab* mutant zebrafish. The significant behavioral differences from wild-type larvae, as demonstrated here, and their sensitivity to pharmacological treatments, expand the repertoire of assays that can be used to characterize zebrafish models for neurologic disease and uncover new treatments.

### Sleep

Evidence from patients, mice, and zebrafish suggest that *SCN1A* homologs play a conserved role in regulating motor activity and daily patterns of locomotion. Zebrafish, including larvae, are diurnal in laboratory conditions, with a light-entrainable circadian clock (Cahill et al., 1998). Wild-type larvae exhibit hallmarks of sleep, including immobility, increased arousal threshold that can rapidly be reversed, characteristic posture, and sleep rebound following deprivation (Zhdanova et al., 2001). Sleep in zebrafish can be quantified as bouts of immobility at night (Elbaz et al., 2013). Our assay simplifies this approach to measure activity levels in day and night without specifying the microstructure of rest bouts. We found disrupted levels of locomotor activity at several points throughout the light-dark cycle, with a consistently increased activity in *scn1lab* ^s552/s552^ larvae compared to wild types during the dark phase. Like humans, and in contrast to mice, zebrafish sleep at night, making them a useful model for diurnal behavioral patterns. High levels of cycling alternating pattern activity in non-REM sleep were reported in DS patients (Dhamija et al., 2014). *Scn1a^+/−^* mice similarly exhibit disrupted circadian activity patterns compared to controls, with decreased activity in the active (dark) phase and increased activity in the rest (light) phase (Han et al., 2012a). Mice with a heterozygous knockin missense *Scn1a* mutation showed increased wakefulness in the active (dark) phase (Papale et al., 2013). Taken together, data from zebrafish and mouse models suggest that diurnal behavioral deficits associated with mutations in *SCN1A* homologs may be an important and conserved feature of *SCN1A* deficiency.

The increased movement that we observed in *scn1lab*^s552/s552^ larvae during the dark phase (night) may correspond directly to the greater electrical signals that were detected during the dark phase using a recently developed microfluidic multielectrode recording chamber method (Hong et al., 2016). Further development and characterization of zebrafish models of epileptic encephalopathies could reveal important mechanistic insights related to sleep. Future *in vivo* imaging and electrophysiology may also contribute to our understanding of neural activity patterns during the course of waking and sleeping (Wang et al., 2011).

### Open field exploration

Our open field assay featured high temporal resolution and revealed severe deficits in open field exploration and movement. Exploration in an open field assay was similarly disrupted in a zebrafish *mecp2* mutant model for Rett syndrome (Pietri et al., 2013). Key features of our assay include the use of six-well plates with 36.6-mm diameter wells, pretreatment with drug for 30 min followed by continuous drug exposure, and immediate recording of locomotion following addition of the larvae to the novel wells. Binning the data into 30-s time bins revealed previously unappreciated features of the *scn1lab* ^s552/s552^ mutant larvae behavior that could have been obscured with larger time bins, including a worsening (decreasing) trend in distance moved over the 5-min assay, accompanied by an improving (increasing) amount of time spent in the center of the arena. In contrast, no evidence was found for habituation in wild-type larvae in a larger (9.6 cm) dish over 15 min (using 5-min time bins; Ahmad and Richardson, 2013). Reduced overall level of movement appears to be the primary effect of clemizole and diazepam in wild-type and mutant larvae, leading to increased variability and differences in duration spent in the center of the arena. Our findings correspond directly to the efficacy of these drugs at this concentration in reducing behavioral measures of seizures, as shown in previous papers. The zebrafish open field test, like the mouse equivalent, has clear limitations as an assay of cognitive function, and should be interpreted with caution as a measure of anxiety since many factors can influence open field behavior (Walsh and Cummins, 1976). Several other cognitive assays have been reported for adult zebrafish (Meshalkina et al., 2017) but are not reliably established for larvae. Overall, our results suggest that modifications to the open field assay were important for revealing behavioral deficits and could be applied to a variety of larval zebrafish mutant models.

### Pharmacology

Valproic acid, diazepam, trazodone, and clemizole have all been studied for antiepileptic activity in *scn1lab* zebrafish larvae (Baraban et al., 2013; Griffin et al., 2017). For these drugs, effects in wild-type zebrafish on diurnal rhythms (Rihel et al., 2010) or other aspects of locomotion (Herrmann, 1993; Richendrfer et al., 2012; Baraban et al., 2013) have also been investigated. Valproic acid, a broad spectrum antiepileptic drug (Tomson et al., 2016) commonly used in DS (Chiron and Dulac, 2011), exerts protective effects in larval or adult zebrafish exposed to the chemoconvulsant pentylenetetrazole: (1) decreasing behavioral or electrographic seizure activity and (2) improving deficits in learning of a passive avoidance response (Lee et al., 2010). Valproic acid has also been shown to increase “waking” activity in wild-type larvae, with a lowest effective dose of 15 μM (Rihel et al., 2010). Although valproic acid at a concentration of 1 mM exerted antiepileptic activity in *scn1lab* ^s552/s552^ mutant larvae (Baraban et al., 2013), no significant effect on behavior was observed here with valproic acid at a concentration of 250 μM. Trazodone, a drug commonly prescribed for insomnia and depression (Rickels et al., 1993; Mendelson, 2005), potentiates the high-speed movements caused by light flash in larval zebrafish (Koseki et al., 2014), and can increase rest (Rihel et al., 2010), but had no significant effects on the behaviors we assayed in the range of concentrations we tested (2.5-250 μM). Diazepam, a benzodiazepine and antiepileptic drug, decreases locomotor activity, seizures, and measures of anxiety in wild-type zebrafish larvae (Zhdanova et al., 2001; Baraban et al., 2005; Schnörr et al., 2012). Diazepam has been tested at a range of concentrations from 10 nM up to 1 mM in larval zebrafish (Zhdanova et al., 2001; Baraban et al., 2005; Richendrfer et al., 2012; Baraban et al., 2013; Griffin et al., 2017). We found that 250 μM diazepam significantly decreased locomotion in an open field test of the *scn1lab* ^s552/s552^ mutant larvae, extending previous results that showed nearly complete elimination of movements at a 100 μM concentration, compared to minimal effects at a 1 μM concentration (Zhdanova et al., 2001; Baraban et al., 2005). An even higher concentration of clemizole (667 μM) led to nearly complete elimination of movement in *scn1lab* ^s552/s552^ mutants following a brief exposure (Baraban et al., 2013). Clemizole, a first-generation antihistamine recently identified as a potential antiepileptic drug acting on serotonin receptors in *scn1lab* ^s552/s552^ mutant larvae (Baraban et al., 2013; Griffin et al., 2017), decreased behavioral activity in the hyperactive *scn1lab* ^s552/s552^ larvae in both the diurnal and open field assays. Clemizole had no significant effects on locomotion at concentrations between 6.25 μM and 50 μM (Baraban et al., 2013). Similarly, clemizole can also increase “rest” activity in wild-type larvae (Rihel et al., 2010), supporting our finding of decreased nighttime locomotion in mutant larvae.

Caffeine, an adenosine receptor antagonist, is thought to produce anxiogenic effects in fish, rodents, and humans. Larval zebrafish exposed to caffeine at 100 mg/l (515 μM) exhibited decreased locomotor activity and reduced swim speed in the open field test (Maximino et al., 2011; Richendrfer et al., 2012). A locomotor depressive effect of high concentrations of caffeine has also been observed in rodents (Finn and Holtzman, 1986; Svenningsson et al., 1995). Our control studies using 250 μM caffeine are consistent with these reports that high concentrations of caffeine will decrease locomotor activity in wild-type larval zebrafish and support a conclusion that features of exploration and anxiety may be conserved between larval zebrafish and mammals.

### Interneuron density

Interneuron defects are thought to be responsible for pathology in DS patients (Yu et al., 2006; Ogiwara et al., 2007; Mistry et al., 2014). Deficits in GABA-mediated inhibition may reflect changes in the number of inhibitory synapses or neurons. Using *in vivo* light sheet microscopy and an interneuron reporter line (i.e., dlx5/6:GFP) we found no difference in the number of GFP-positive neurons in *scn1lab* ^s552/s552^ mutant larvae and control siblings. As these GFP cells are primarily GABAergic interneurons (Robles et al., 2011), our data suggest that interneurons are present in normal abundance at these early stages of development. Consistent with our findings in a zebrafish model, differences in interneuron density in mouse models of *Scn1a* deficiency, or patients with DS, have not been reported.

## Conclusion

Some of the greatest advantages of *in vivo* disease modeling using larval zebrafish are the ease of genetic modifications (Varshney et al., 2013; Gagnon et al., 2014; Li et al., 2016), the broad range of behavioral assays available (Brockerhoff et al., 1995; Budick and O'Malley, 2000; Fero et al., 2011), and the scalability for phenotype-based drug screening (Rihel et al., 2010; Gut et al., 2013; Dinday and Baraban, 2015; Bruni et al., 2016). As demonstrated here, clinically relevant comorbidities such as sleep, movement disorders, and anxiety can be efficiently assayed in a larval zebrafish model of DS, despite concerns that “lower model organisms” such as zebrafish would not allow assessment of comorbid symptoms (EpiPM Consortium, 2015). Moreover, these assays combined with the unique attributes of larval zebrafish for higher throughput drug screening (e.g., large clutch sizes, multi-well readouts, and small-molecule permeation), can be used for the rapid identification of drugs that reduce behavioral deficits.

*Note Added in Proof:* The commercial interest was accidentally left off the title page of the Early Release version published August 3, 2017. The commercial interest has now been included in the “Disclosures” section.

## Synthesis

Reviewing Editor: Alfonso Represa, INSERM Decisions are customarily a result of the Reviewing Editor and the peer reviewers coming together and discussing their recommendations until a consensus is reached. When revisions are invited, a fact-based synthesis statement explaining their decision and outlining what is needed to prepare a revision will be listed below. The following reviewer(s) agreed to reveal their identity: Gerald Downes.

Dear authors,

Both reviewers evaluated again you revised version and appreciated the improvements. They are still supportive of your report and recognize the interest of your data. There are however a few points that would require revision and that you'll find described below. Note that both referees wonder if the interpretations of data from open field tests, mainly on the pharmacologic effects, are convincing and make some suggestions for clarifying the issue. I think this is important to do.

Referee 1:

1) The limits of the model should be clearly stated (also in the abstract) and discussed: this zebrafish model can be used to reproduce some of the behavioral comorbidities associated with Dravet syndrome, but it has clear limits as a model of other comorbidities (e.g. cognitive defects).

2) From the data in fig 4, it seems that with diazepam & clemizole fish basically don't move (in particular with diazepam): they stay in the center without moving... Is this a real amelioration? Moreover, in the open field Wt fish move more than mutant ones. This defect is amplified by diazepam or clemizole...

3) For the statistical analysis of the data shown in fig 2 and table 1, ANOVA or a non parametric equivalent should be used. Which test has been used to check normality and equality of variance (e.g. Welch t-test has been used in Fig 1, fig 3 but not for Table 1

4) In their response, the authors stated:

‘Locomotion tracking software automatically detects larvae that are darker than the background in the DanioVision system (Noldus). We have two DanioVision systems in the lab and the older system circa 2012 (which was used for the initial light-dark studies presented in Figure 1) has a camera that appears to be less sensitive than the newer DanioVision system circa 2016 (used for all subsequent studies). Thus, the absolute values in mm for distance moved are different between DanioVision systems and not directly comparable.’

This should be clearly described in the methods section

5) Results p8 line 215-216. The time with and without DMSO should be reported

Referee 2:

6) Given that the authors state that there are new features of their open field assay compared to previously published data, the wild-type responses to different drugs should be shown in this new context. Also, for the reader's convenience, the authors should plot the wild-type activity obtained in Figure 3 in Figure 4. This would facilitate evaluation of whether drugs restore mutant activity to wild-type levels.

7) The decreased locomotion reported in the open field pharmacology experiments seem to preclude meaningful conclusions about anxiety. With increased anxiolytic effects alone, you would expect to observe increased exploratory behavior along with increased time in the center. The data shown here are only decreases in locomotor behavior and the increased time in the center could be incidental to the starting location. For example, would a deeply anesthetized or dead larva provide a different result than the 250uM diazepam results? What would the diazepam data look like if the larvae started out at the edge of the well? The authors could address this concern by taking into account the starting positions of the larvae in the well and/or, for comparison purposes to the drugs they used, testing drugs have been shown to regulate anxiety and locomotion separately.

8) The concentration of diazepam utilized to reveal an effect in the open field pharmacology experiments seems too high. 250uM is several fold higher than that used in at least a couple previous studies which describe its anxiolytic effects in larval zebrafish (for example, Richendrfer et al. 2012). Intermediate concentration(s) of should be used.

9) Minor correction: -The orientation and abbreviations used in Figure 5 should be articulated in the figure legend.
